# Supplementary material for: A computational program for automated surgical planning of fenestrated endovascular repair
Source: Commun Eng. 2023 Jun 13;2:37. doi: 10.1038/s44172-023-00083-2 (PMC10955905; doi:10.1038/s44172-023-00083-2)
Supplement: Supplementary file 2 — Supplementary Information [file 44172_2023_83_MOESM2_ESM.pdf]

A Computational Program for Automated Surgical Planning of Fenestrated Endovascular Repair - *Supplementary Information*

Tom M. Dillon[1], Patric Liang[2], Marc L. Schermerhorn[2], Ellen T. Roche[1,3]

1. Department of Mechanical Engineering, Massachusetts Institute of Technology; 77 Massachusetts Avenue, Cambridge, MA 02139
2. Department of Surgery, Division of Vascular and Endovascular Surgery, Beth Israel Deaconess Medical Center and Harvard Medical School; 330 Brookline Avenue, Boston, MA 02215
3. Institute for Medical Engineering and Science, Massachusetts Institute of Technology; 77 Massachusetts Avenue, Cambridge, MA 02139

Corresponding Author:

Ellen T. Roche

77 Massachusetts Avenue, E25-344
Cambridge, MA 02139
Telephone: 617-258-6024
E-mail: etr@mit.edu

Supplementary Methods

S1 Uniform Graft Mask Generation

Initially a binary template is generated for the graft mask, where ‘1’ represents a pixel containing a strut, and ‘0’ represents a free area of the graft where fenestrations can be placed. FenFit contains a flexible design repository which allows the user to represent an infinite number of graft templates. The user only needs to input a few key design parameters to represent an arbitrary graft in the virtual space:

1. Stent strut wavelength – λ
2. Stent strut amplitude – A
3. Number of stent rings – N
4. Graft diameter - D
5. Graft length – L
6. Gap length – GL

The footprint of the flattened cylindrical graft is a rectangle of size $(\pi D) \times L$. A gap of GL spaces each cosine wave. Simple cosine functions are used to generate the stent struts. There are an infinite number of locations the fenestrations can be aligned on the graft, though computationally, it is only feasible to search across a finite number of these configurations. Therefore, the graft is discretized into a 2D array of pixels.

S2 Tapered Graft Cylindrical-Planar Mapping

Tapered grafts map discretely to 3 distinct segments, as illustrated in **Figure S1**. While the basic search functionality of the program is the same for tapered grafts as for uniform grafts, the 3D to 2D mapping transformation differs for each design. The uniform proximal and distal portions of the graft are mapped as separate uniform diameter grafts, where scaling is applied to account for their varying diameters.

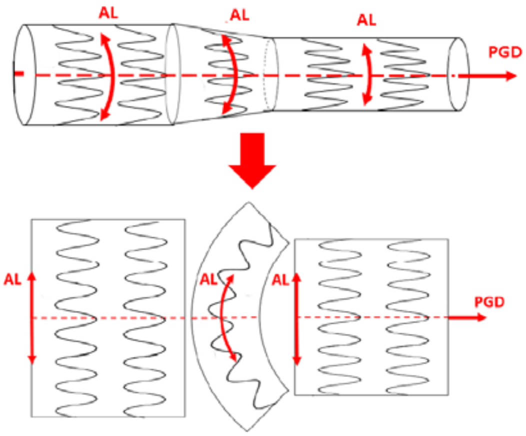

Figure S1 – Generation of a *tapered* fenestration mask. The proximal and distal portions of the graft map to rectangles of varying outer dimensions, while the tapered portion of the graft can be approximated as a truncated cone in 3D that maps to a truncated annulus in 2D.

The tapered portion of the graft is treated as a truncated cone geometry, which in turn maps to a truncated circle sector in 2D. The 2D coordinate system can be split into 3 discrete portions for implementation in FenFit (depicted in **Figure S2**).

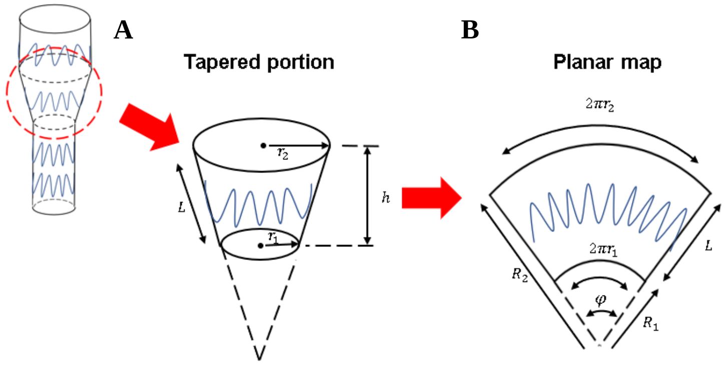

Figure S2 – Parameterization of truncated geometry (a) Truncated cone geometry and relevant geometric parameters for generating tapered graft templates. (b) Flattened representation of tapered graft design.

For the tapered portion, fenestrations do not move parallel to the *AL-PGD* axes as the graft is moved axially and rotationally. The dimensions of the truncated sector can be determined using trigonometry based on the graft parameters defined in **Figure S2**:

$$L = \sqrt{(r_2 - r_1)^2 + h^2}$$

$$R_1 = \frac{L r_1}{r_1 - r_2}$$

$$R_2 = L + R_1$$

$$\phi = 360 * \frac{r_2}{R_2}$$

Where r_1 and r_2 are the large and small diameters of the graft respectively, and h is the vertical length of the tapered portion. The tapered stent struts can then be projected to 2D using a product of sines and cosines:

$$strut_x(t) = C_x + \left[(R_{base} + A)\, cos\, cos\, \frac{2\pi qt}{\phi}\right] cos\ (t)$$

$$strut_y(t) = C_y + \left[(R_{base} + A)\, sin\, sin\, \frac{2\pi qt}{\phi}\right] sin\ (t)$$

Where C is the center of curvature, R_{base} is the zero amplitude-reference of the strut interpolated between R_1 and R_2, and q is the number of struts per circumference. By the

equation above, movement of the fenestrations in the arclength direction will be curved on the tapered portion of the graft.

S2 CT Measurement and Fenestration Mask Generation

We define the proximal graft distance (*PGD*) as the distance between a fenestration and the proximal end of the graft, and arclength (*AL*) as the circumferential position of each fenestration along the graft's surface. To generate the fenestration mask, the fenestration positions must be projected to 2D based on their 3D location in the aortic CT scan. The sagittal basis vector, s (a reference vector obtained from the CT scan), is projected to each cross section of the aorta to act as a reference for measuring fenestration angles. This vector is the orthogonal projection of s on n in **Figure S3** (also known as the vector rejection [1]) and is calculated by,

$$b_1 = s - proj_s n - \frac{s \cdot n}{n \cdot n} n$$

Where b_1 is the first basis vector on the profile plane. The other basis vector, b_2, is calculated via the cross product of n and b_1. b_2 is later used to generate a circular cross section of the graft suitable for lofting.

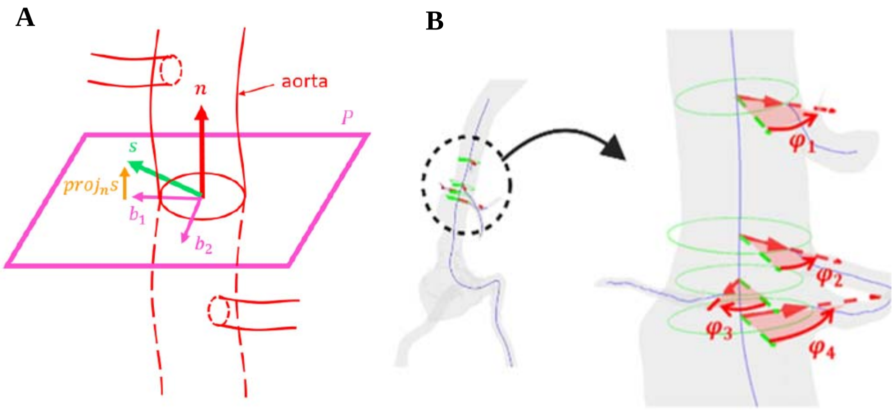

Figure S3 –Calculation of fenestration angles. (a) illustration of the relevant variables, where the normal vector (*n*), sagittal vector (*s*), and basis vectors (b_1 and b_2) are indicated for a single aortic cross section. (b) Fenestration angles for a sample CT scan.

Finally, the AL measurements can be calculated using the formula below,

$$AL = |r|cos^{-1}(\varphi) = |r|cos^{-1}\left(\frac{b_1 \cdot r}{r|r|}\right)$$

Where r represents the vector between the fenestration and its closest point along the aortic centreline. This vector can be calculated assuming we know the 3D position of each fenestration using existing automated centreline extraction tools (e.g. 3D Slicer [2]). Finally, *PGD* is measured as the distance of the cross section along the aorta. The footprint for the fenestration mask is the same as that of the graft mask (dimensions of $(\pi D) \times L$). Given we now have measurements for both *AL* and *PGD,* the fenestrations can be generated by setting all pixels within a radius of R of the fenestration centres to a value of '1'.

S4 Revised Search Strategy:

To maximize the probability of obtaining a valid fit, FenFit can employ alternative search strategies. The two primary search techniques are as follows:

• **A favored search** – this is the first search conducted, which identifies graft configurations where no overlap exists between the stent struts and the fenestrations.

• **A revised search** – A relaxed search is conducted only when a favored search fails, facilitating independent adjustment of the left and right renal arteries, as well as relaxation of the prioritisation level of vessels.

During a revised search, the fit for some vessels may be prioritized over others. There are 3 distinct prioritization levels, which were informed by the preferences of our clinical collaborators at Beth Israel Deaconess Medical Center, Boston: (1) a priority vessel, where no overlap is permitted between the fenestrations and the stent struts, (2) A non-priority vessel,

where some overlap may be allowed, and (3) left and right renal artery adjustment, where the fenestrations may undergo up to 3mm posterior movement in the posterior *AL* direction (towards the patient's back) to achieve a valid fit. This additional degree of freedom is highlighted in **Figure S4**. FenFit will select the minimal posterior adjustment of the renal fenestrations that obtains an overall valid fit. The program recommended a fit that included non-prioritized vessels for 2 patients from the 25-patient cohort (see **Table S1**).

The program also allows the physician to manually adjust the patient geometry by clicking and dragging the fenestration independently if desired. Overall, the techniques described above increase the likelihood of locating a valid fit by using modified search criteria.

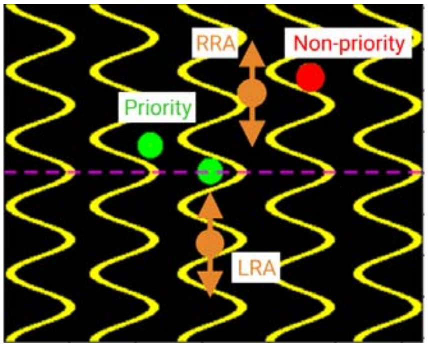

Figure S4 - Fenestration degrees of freedom during the revised search regime. No overlap is permitted in the case of the prioritised fenestrations (green), though the non-priority vessels do facilitate some overlap (red). The left and right renal arteries each have a degree of freedom to move independently of the other fenestrations in the arclength direction (orange).

S5 3D Graft Mesh Generation

Prior to mesh parameterization and texture mapping, a graft mesh is generated inside the aorta to represent the template geometry. As mentioned previously, the initial aortic centreline is extracted using automated centreline tools in 3D slicer. A spline of best fit is used to smooth

the noisy centreline data obtained from the CT scan, and is defined as the curve that minimizes the optimization function:

$$f = (U_i - \hat{f}(x))^2 + \lambda \int f''(x)^2 dx$$

Where U are the coordinates of the aortic centreline, $\hat{f}$ is the smoothing spline, and λ is the second derivative roughness parameter.

Profile normals at the top and bottom of the graft were defined to determine an initial sweep direction. Finally, the graft profile is swept through the spline of best fit to obtain the desired template geometry. The final geometry can also be varied based depending on whether a uniform or tapered graft is selected by the surgeon. The overall graft mesh generation workflow is summarized in Figure S5.

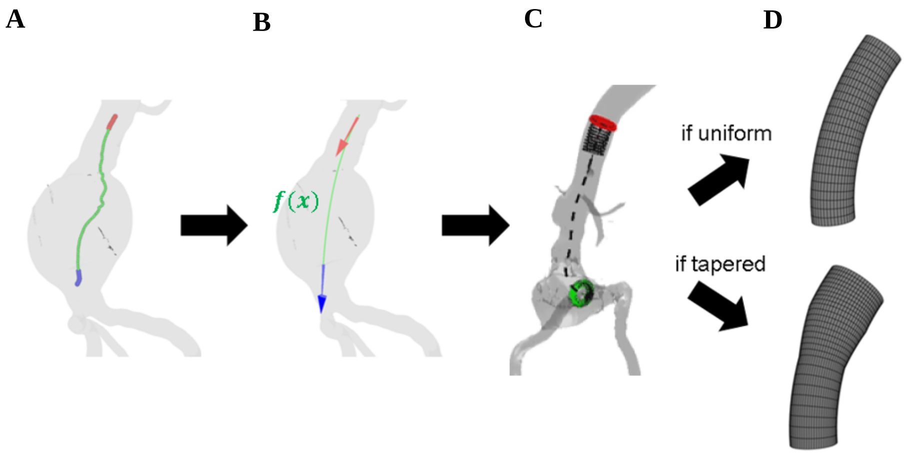

Figure S5 The graft mesh generation workflow consists of; centreline extraction from the CT scan (a), smoothing using an optimized spline (b), and lofting the mesh along the spline of best fit (c) to yield a final template design (d).

S6 User Interface Design

A key requirement for FenFit was to develop a program that provides an easy-to-use, intuitive, user interface (UI). The four stage UI for FenFit is highlighted in **Figure S6** and Supplementary **Video SV 1**. When the user first enters the program, they are prompted to select a graft template design from a repository of commercially available endografts. The user can preview the corresponding graft mask for each design to ensure the dimensions of the template are accurate before proceeding. On the second panel, the user inputs the patient's anatomy (in the form of an aortic segmentation), which allows rendering of a "skeleton model" of the aortic and fenestration centrelines on screen. If a segmentation is not available, the physician can manually input *AL* and *PGD* measurements to the UI. The user may visualize the search algorithm for verification purposes or hide this step for more efficient computation. The final panel provides the primary results of the program – FenFit reports the placement accuracy of each fenestration and allows the user to inspect the graft and fenestration positions relative to the CT scan. A word document is also compiled summarizing the main results, as well as instructions for graft modification in the operating room.

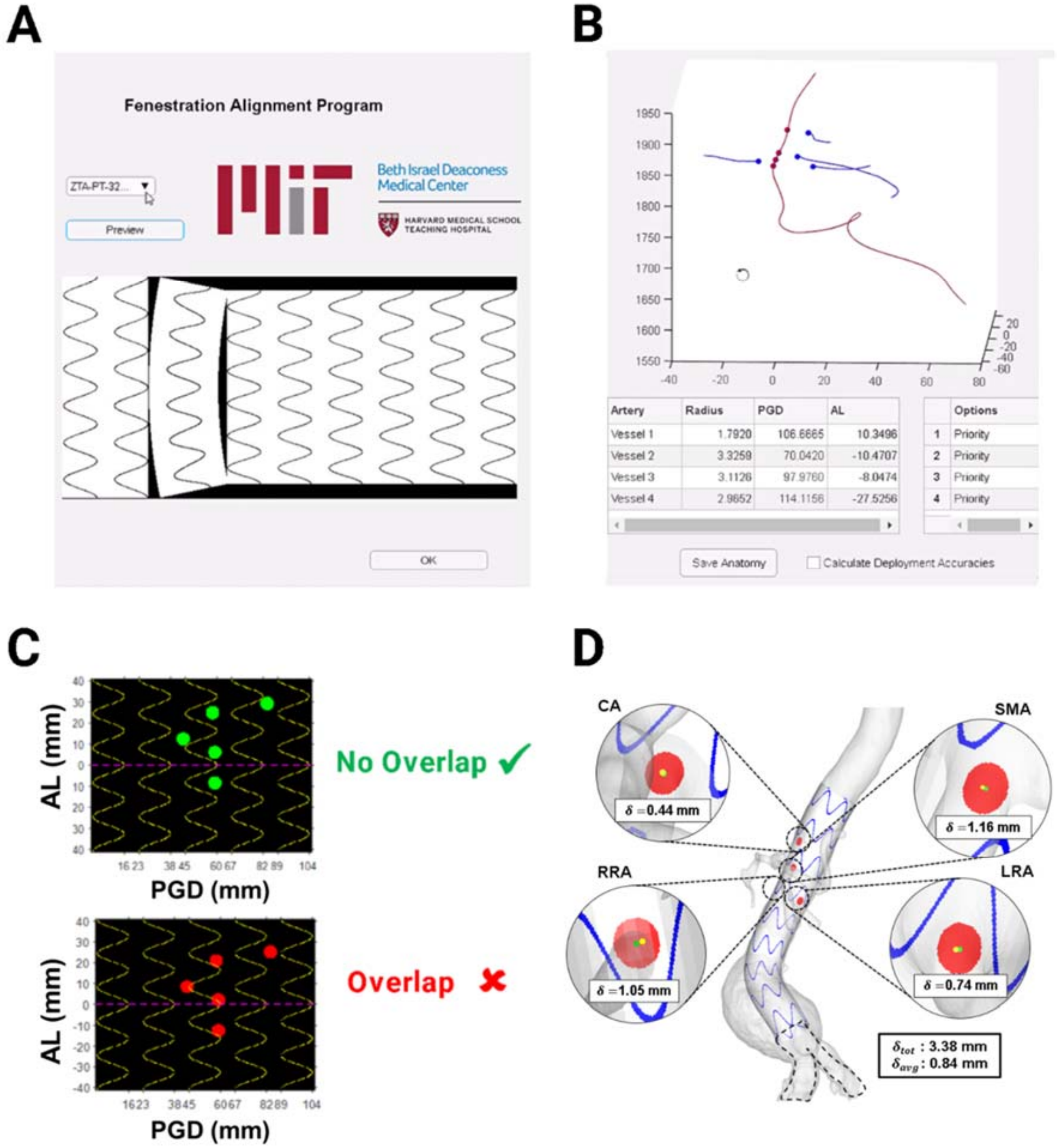

Figure S6 User interface workflow in FenFit software. The physician first selects a graft template from a user-defined repository (A) A "skeleton" of the aortic centreline (red) and fenestration centrelines (blue) are extracted automatically from the CT scan (B) The user can choose to visualize the search process for verification purposes (C) Finally, the results from the search process are visualized, and the user can export a report if desired (D).

S7 Summary of Demographic Data and Program Results

Table S1 Clinical characteristics and comparison of fenestration planning time for 25 abdominal aneurysm cases using manual physician modified endograft planning against automated FenFit approach.

ID	Age	Sex	FenFit plan time (min)	Manual plan time (min)	Non-prioritized vessels?
1	73	M	0.3	15.6	-
2	79	M	2.1	14.0	-
3	75	M	2.6	25.5	-
4	62	M	0.4	21.1	-
5	0	F	1	23.6	-
6	72	M	2.1	19.0	-
7	66	M	0.5	15.0	-
8	79	M	2.1	14.0	-
9	78	F	0.3	22.1	-
10	80	F	0.9	28.3	-
11	70	M	0.4	23.0	-
12	78	M	0.3	22.1	-
13	82	M	0.4	32.0	-
14	62	M	0.4	21.1	-
15	69	M	1.5	16.1	-
16	63	F	0.3	34.0	-
17	81	F	2.1	18.0	-
18	71	M	0.3	17.1	-
19	81	M	3.4	26.0	-
20	73	M	0.2	26.2	-
21	75	F	1.3	25.4	Yes
22	81	M	1.0	21.3	Yes
23	78	F	0.3	36.5	-
24	60	M	0.6	32.0	-
25	85	M	0.4	26.0	-

Table S2 Comparison of fenestration deviation for 10 abdominal aneurysm cases using automated FenFit approach against manually measured physician AL and PGD measurements.

FenFit accuracies				Physician Accuracies			
**C**	**SMA**	**LRA**	**RRA**	**C**	**SMA**	**LRA**	**RRA**
1.01	1.24	2.61	1.36	3.15	2.01	1.72	3.97
0.8	0.9	0.99	0.6	5.13	5.96	4.948	2.82
1.09	0.46	0.67	0.75	2.15	3.37	3.42	0.39
1.16	0.44	0.74	1.05	1.86	4.46	3.5	2.27
1.16	2.38	1.15	1.05	0.9	2.05	2.66	1.65
0.69	1.3	0.99	0.97	2.09	2.72	2.36	3.6
1.46	0.57	1.35	0.6	1.35	8.32	3.01	7.87
0.24	0.49	1.65	0.52	1.74	0.5	1.94	3.56
0.95	0.91	0.59	0.111	1.75	3.68	1.48	1.83
0.07	0.48	0.25	0.53	2.04	2.01	0.66	3.87

Supplementary References

[1] F.S. Hill Jr., "Graphics Gems IV," *San Diego: Academic Press.,* pp. 138–148, 1994

[2] A. Fedorov, R. Beichel, J. Kalpathy-Cramer, J. Finet, J-C. Fillion-Robin, S. Pujol, C. Bauer, D. Jennings, F.M. Fennessy, M. Sonka, J. Buatti, S.R. Aylward, J.V. Miller, S. Pieper, R. Kikinis, "3D Slicer as an Image Computing Platform for the Quantitative Imaging Network," *Magn Reson Imaging.*, vol. 9, pp. 1323-41, 2012.
