# Supplementary material for: A computational program for automated surgical planning of fenestrated endovascular repair
Source: Commun Eng. 2023 Jun 13;2:37. doi: 10.1038/s44172-023-00083-2 (PMC10955905; doi:10.1038/s44172-023-00083-2)
Supplement: Supplementary file 3 — Description of Additional Supplementary Files [file 44172_2023_83_MOESM3_ESM.pdf]

# Description of Additional Supplementary File

**File name:** Supplementary Video SV 1

**Description:** The four stage UI for FenFit is highlighted in Supplementary Video SV 1. When the user first enters the program, they are prompted to select a graft template design from a repository of commercially available endografts. The user can preview the corresponding graft mask for each design to ensure the dimensions of the template are accurate before proceeding. On the second panel, the user inputs the patient’s anatomy (in the form of an aortic segmentation), which allows rendering of a “skeleton model” of the aortic and fenestration centrelines on screen. If a segmentation is not available, the physician can manually input AL and PGD measurements to the UI. The user may visualize the search algorithm for verification purposes or hide this step for more efficient computation. The final panel provides the primary results of the program – FenFit reports the placement accuracy of each fenestration and allows the user to inspect the graft and fenestration positions relative to the CT scan. A word document is also compiled summarizing the main results, as well as instructions for graft modification in the operating room.
