# Supplementary material for: A computational program for automated surgical planning of fenestrated endovascular repair
Source: Commun Eng. 2023 Jun 13;2:37. doi: 10.1038/s44172-023-00083-2 (PMC10955905; doi:10.1038/s44172-023-00083-2)
Supplement: Supplementary file 5 — Reporting Summary [file 44172_2023_83_MOESM5_ESM.pdf]

nature portfolio

Corresponding author(s): Ellen Roche

Last updated by author(s): Apr 29, 2023

Reporting Summary

Nature Portfolio wishes to improve the reproducibility of the work that we publish. This form provides structure for consistency and transparency in reporting. For further information on Nature Portfolio policies, see our Editorial Policies and the Editorial Policy Checklist.

Statistics

For all statistical analyses, confirm that the following items are present in the figure legend, table legend, main text, or Methods section.

n/a	Confirmed	
☐	☒	The exact sample size (*n*) for each experimental group/condition, given as a discrete number and unit of measurement
☐	☒	A statement on whether measurements were taken from distinct samples or whether the same sample was measured repeatedly
☐	☒	The statistical test(s) used AND whether they are one- or two-sided<br>*Only common tests should be described solely by name; describe more complex techniques in the Methods section.*
☐	☒	A description of all covariates tested
☐	☒	A description of any assumptions or corrections, such as tests of normality and adjustment for multiple comparisons
☐	☒	A full description of the statistical parameters including central tendency (e.g. means) or other basic estimates (e.g. regression coefficient) AND variation (e.g. standard deviation) or associated estimates of uncertainty (e.g. confidence intervals)
☐	☒	For null hypothesis testing, the test statistic (e.g. *F*, *t*, *r*) with confidence intervals, effect sizes, degrees of freedom and *P* value noted<br>*Give P values as exact values whenever suitable.*
☒	☐	For Bayesian analysis, information on the choice of priors and Markov chain Monte Carlo settings
☒	☐	For hierarchical and complex designs, identification of the appropriate level for tests and full reporting of outcomes
☒	☐	Estimates of effect sizes (e.g. Cohen's *d*, Pearson's *r*), indicating how they were calculated

Our web collection on statistics for biologists contains articles on many of the points above.

Software and code

Policy information about availability of computer code

Data collection	Given the authors have filed a patent application related to this work (U.S. Patent application number PCT/US2021/060591), the custom code implemented in this manuscript is not available to the public at the time of writing. In the event of patent publication, the code will be available upon reasonable request from the authors. No commercial or open source code was used in this work.
Data analysis	Given the authors have filed a patent application related to this work (U.S. Patent application number PCT/US2021/060591), the custom code implemented in this manuscript is not available to the public at the time of writing. In the event of patent publication, the code will be available upon reasonable request from the authors. No commercial or open source code was used in this work.

For manuscripts utilizing custom algorithms or software that are central to the research but not yet described in published literature, software must be made available to editors and reviewers. We strongly encourage code deposition in a community repository (e.g. GitHub). See the Nature Portfolio guidelines for submitting code & software for further information.

Data

Policy information about availability of data

All manuscripts must include a data availability statement. This statement should provide the following information, where applicable:

- Accession codes, unique identifiers, or web links for publicly available datasets
- A description of any restrictions on data availability
- For clinical datasets or third party data, please ensure that the statement adheres to our policy

We have made the following data publicly available on figshare [16]:
• De-identified patient CT segmentations fed as input to the FenFit program.
• Raw data related to planning times and planning accuracy collected by surgeons at Beth Israel Deaconness Medical Centre, Boston.
• Beth Israel Deaconness Medical Center Boston (BIDMC) FenFit Study Protocol

Human research participants

Policy information about studies involving human research participants and Sex and Gender in Research.

Reporting on sex and gender	N/A
Population characteristics	N/A
Recruitment	N/A
Ethics oversight	N/A

Note that full information on the approval of the study protocol must also be provided in the manuscript.

Field-specific reporting

Please select the one below that is the best fit for your research. If you are not sure, read the appropriate sections before making your selection.

☒ Life sciences ☐ Behavioural & social sciences ☐ Ecological, evolutionary & environmental sciences

For a reference copy of the document with all sections, see nature.com/documents/nr-reporting-summary-flat.pdf

Life sciences study design

All studies must disclose on these points even when the disclosure is negative.

Sample size	A single-institution retrospective review of anonymized abdominal computed tomography (CT) scans in all consecutive patients scheduled to undergo fenestrated aortic endovascular repair at Beth Israel Deaconess Medical Centre (BIDMC) from August 2020 to January 2021 was performed to evaluate the efficiency and accuracy of FenFit compared to physician manual planning. Visceral anatomy was segmented for 10 CT scans from the 25-patient cohort, allowing the alignment deviation to be calculated for those cases. The remaining 15 scans were not taken at the appropriate level of contrast necessary for fenestration segmentation, and therefore only the planning time (and not the alignment deviation) was measured in these cases.
Data exclusions	As stated in manuscript: Visceral anatomy was segmented for 10 CT scans from the 25-patient cohort, allowing the alignment deviation to be calculated for those cases. The remaining 15 scans were not taken at the appropriate level of contrast necessary for fenestration segmentation, and therefore only the planning time (and not the alignment deviation) was measured in these cases.
Replication	The planning algorithm is deterministic - therefore we would expect our results (timing and accuracy) to be reproducible for a given set of inputs (CT scans).
Randomization	As stated in manuscript: Planning times were recorded by the surgeon for 25 retrospective CT scans using the automated FenFit approach and traditional physician manual planning. FenFit planning and manual planning were run independently using the same fenestration AL and PGD data from the patient CT scan as input, akin to the parallel workflows illustrated in Figure 1C. Six vascular fellows of various aortic planning experience, ranging from 0 to 3 years took part in the study. A single fellow was randomly assigned to each of the clinical cases, performed the calculations, and found the fenestration fits independently. The FenFit algorithm also selected an optimal graft design independent from the fit manually located by the physician.
Blinding	As stated in the manuscript, it should be noted that only a single vascular fellow was assigned for manual planning of each case. In our future work, we will include multiple vascular surgeons per case, to better understand the variability in case planning times that may arise amongst physicians. Surgeons were not cross-assigned in this study due to limited time they had to participate in the study.

Reporting for specific materials, systems and methods

We require information from authors about some types of materials, experimental systems and methods used in many studies. Here, indicate whether each material, system or method listed is relevant to your study. If you are not sure if a list item applies to your research, read the appropriate section before selecting a response.

Materials & experimental systems

n/a	Involved in the study
☒	☐ Antibodies
☒	☐ Eukaryotic cell lines
☒	☐ Palaeontology and archaeology
☒	☐ Animals and other organisms
☐	☒ Clinical data
☒	☐ Dual use research of concern

Methods

n/a	Involved in the study
☒	☐ ChIP-seq
☒	☐ Flow cytometry
☒	☐ MRI-based neuroimaging

Clinical data

Policy information about clinical studies

All manuscripts should comply with the ICMJE guidelines for publication of clinical research and a completed CONSORT checklist must be included with all submissions.

Clinical trial registration	FDA-IDE, clinical trial registration number: NCT04746677
Study protocol	Available on Figshare - see data availability statement above
Data collection	A single-institution retrospective review of anonymized abdominal computed tomography (CT) scans in all consecutive patients scheduled to undergo fenestrated aortic endovascular repair at Beth Israel Deaconess Medical Centre (BIDMC) from August 2020 to January 2021 was performed to evaluate the efficiency and accuracy of FenFit compared to physician manual planning
Outcomes	As detailed in manuscript: The two primary outputs from FenFit are graft modification instructions on where to cauterize the fenestrations on the endograft in the OR, as well as a 3D overlay of the final fenestrated graft design alongside the aortic segmentation. In addition to these outputs, the user is given an estimate for the placement accuracy of the FenFit program.
